# Distributional regression modeling via generalized additive models for location, scale, and shape: An overview through a data set from learning analytics

**DOI:** 10.1002/widm.1479

**Published:** 2022-10-21

**Authors:** Fernando Marmolejo‐Ramos, Mauricio Tejo, Marek Brabec, Jakub Kuzilek, Srecko Joksimovic, Vitomir Kovanovic, Jorge González, Thomas Kneib, Peter Bühlmann, Lucas Kook, Guillermo Briseño‐Sánchez, Raydonal Ospina

**Affiliations:** ^1^ Centre for Change and Complexity in Learning University of South Australia Adelaide Australia; ^2^ Instituto de Estadística Universidad de Valparaíso Valparaíso Chile; ^3^ Department of Statistical Modelling Institute of Computer Science of the Czech Academy of Sciences Prague Czech Republic; ^4^ Czech Institute of Informatics Robotics and Cybernetics, CTU Prague Czech Republic; ^5^ Computer Science Education/Computer Science and Society Research Group Humboldt University of Berlin Berlin Germany; ^6^ Departamento de Estadística Pontificia Universidad Católica de Chile Santiago de Chile Chile; ^7^ Campus Institute Data Science (CIDAS) and Chair of Statistics Georg‐August‐Universität Göttingen Göttingen Germany; ^8^ Seminar for Statistics, ETH Zürich Zürich Switzerland; ^9^ Epidemiology, Biostatistics, and Prevention Institute University of Zurich Zurich Switzerland; ^10^ Institute of Data Analysis and Process Design Zurich University of Applied Sciences Winterthur Switzerland; ^11^ Department of Statistics TU Dortmund University Dortmund Germany; ^12^ Department of Statistics, CASTLab Federal University of Pernambuco Recife Brazil

**Keywords:** causal regularization, causality, educational data mining, generalized additive models for location, scale, and shape, learning analytics, machine learning, statistical learning, statistical modeling, supervised learning

## Abstract

The advent of technological developments is allowing to gather large amounts of data in several research fields. Learning analytics (LA)/educational data mining has access to big observational unstructured data captured from educational settings and relies mostly on unsupervised machine learning (ML) algorithms to make sense of such type of data. Generalized additive models for location, scale, and shape (GAMLSS) are a supervised statistical learning framework that allows modeling all the parameters of the distribution of the response variable with respect to the explanatory variables. This article overviews the power and flexibility of GAMLSS in relation to some ML techniques. Also, GAMLSS' capability to be tailored toward causality via causal regularization is briefly commented. This overview is illustrated via a data set from the field of LA.

This article is categorized under:

Application Areas > Education and Learning
Algorithmic Development > Statistics
Technologies > Machine Learning

## Graphical abstract

Performance of GAMLSS against some machine learning algorithms.

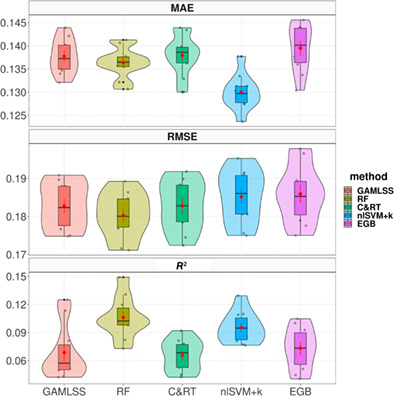

## 1 INTRODUCTION

Most of the data in the field of learning analytics (LA) and educational data mining (EDM) are characterized by being big, second‐hand, observational, and unstructured (Motz et al., 2018).^1^ The data are big because they come from physical and virtual educational environments with many instructors and thousands of students and for whom several metrics exist (e.g., number of clicks, time stamps, course grades, etc.). The data are second‐hand, observational, and unstructured because they are not obtained directly and the type and number of variables are not controlled by the researcher. Although such type of data are amenable to post hoc analyses only and do not allow confident causal inference (i.e. only experimentation enables so by securing first‐hand and structured data; see Imai et al., 2008, 2013), they are nonetheless rich and should be statistically treated to extract valuable practical information.

After gathering educational data, the LA/EDM analytical pipeline begins with preprocessing the data so it is amenable to subsequent statistical treatment. Data preprocessing consumes more than 50% of the pipeline and, among other things, it implies selecting and transforming variables of interest (Romero & Ventura, 2020). Given a large chunk of the analytical pipeline is spend on data preprocessing, it is no surprise that unsupervised learning algorithms are heavily relied on in order to make sense of the data (Joksimovic et al., 2018; see also chapter 5 in Brooks & Thompson, 2017). Those algorithms are also used simply because there is no clear dependent variable. Advances in machine learning (ML), however, enable to submit data with no clear dependent variable to a set of unsupervised algorithms (e.g., clustering algorithms). Although unsupervised learning algorithms can meet their intended goal, they somewhat minimize human decision‐making in the process of statistical model building. Supervised modeling and ML, on the other hand, are clearly targeted for a response variable of interest (e.g., as for targeted learning [a.k.a, superlearner]; see Van der Laan, 2017).

Once an explanatory model has been identified in the analytical pipeline, it is then tested for its predictive power (e.g., via cross‐validation; see Yu & Kumbier, 2020 for a proposal of the place of cross‐validation in the analytic pipeline). More importantly, practitioners are mostly in need of interpretable models that can even license causal interpretations. This article has the goal of providing an overview of generalized additive models for location, scale, and shape (GAMLSS); a supervised distributional regression framework that promotes statistical modeling of entire conditional distributions rather than conditional means. It is also argued that such a framework indeed allows for more causal‐oriented interpretation and better external validity. The outline of this article is as follows: first, a brief technical review of GAMLSS are provided; second, an LA data set is described; third, the LA data are modeled via GAMLSS; and fourth, a link between GAMLSS and causal regularization is proposed. The discussion section considers distributional regression modeling in the larger context of statistical learning. Related GAMLSS‐based analyses such as gradient‐based boosting GAMLSS and penalized GAM are provided as Supporting Information along with their R codes at https://cutt.ly/2WuyxXz.

## 2 GAMLSS AS A DISTRIBUTIONAL REGRESSION FRAMEWORK FOR STATISTICAL LEARNING

One of the traditional preprocessing practices in LA/EDM research consists of discretising continuous variables in order to enhance their interpretability (see section 3.3 in Romero & Ventura, 2020). Slicing a uniform or normally distributed continuous variable in three quantile‐based bins (i.e., high, medium, and low) has been shown to approximate quite well a linear regression (Gelman & Park, 2008). However, in practice, and particularly in the social sciences and education fields, continuous variables tend to follow non‐normal shapes (Bono et al., 2017). This fact then suggests that traditional regression models are not optimal and slicing numeric variables will give biased results (see Bennette & Vickers, 2012 for an example of how categorization of continuous data in epidemiology leads to biased estimation). Hence, flexible and interpretable regression techniques are needed to model such type of data. GAMLSS are a regression framework that enables performing comprehensive statistical learning on the distribution of the response variable with respect to the covariates.

GAMLSS are a class of supervised learning tools for semi‐parametric regression problems that have led to a growing sophistication in the ML field. From a strict statistical modeling viewpoint (McCullagh, 2002), GAMLSS are used to analyze nonlinear relationships between the distributions of outcomes and covariates (features, in ML parlance) and where the covariates' effects are additively weighted.^2^ These models were proposed by Rigby and Stasinopoulos (2001), Akantziliotou et al. (2002), and Rigby and Stasinopoulos (2005) as an improvement and extension to the generalized linear models (GLM) (McCulloch, 2000; Nelder & Wedderburn, 1972) and the GAM (Hastie & Tibshirani, 1990). Key to GAMLSS are that they enable data analyses that exhibit parsimony, generality, consilience, and predictive capacity (Friedman & Silverman, 1989; Picard & Cook, 1984).

GAMLSS have been used in several fields including high‐dimensional regression (De Bastiani et al., 2018; Groll et al., 2019; Hofner et al., 2016; Mayr et al., 2012), psychometrics (Timmerman et al., 2021), neuroimaging (Bethlehem et al., 2022), vision research (Truckenbrod et al., 2020), ecology (Smith et al., 2019), economics (Hohberg et al., 2020), linguistics (Coupé, 2018), hydrology (Dabele et al., 2017), survival analysis (De Castro et al., 2010), clinical management of hearing loss (Hu et al., 2015), insurance (Gilchrist et al., 2009), real‐state appraisal of land lots (Florencio et al., 2012), film box‐office revenues (Voudouris et al., 2012), among others, and just recently GAMLSS have been proposed as new statistical tool for psychological research (Campitelli et al., 2017). Software‐wise, GAMLSS are implemented in R through the gamlss package (Rigby et al., 2020; Stasinopoulos & Rigby, 2007; Stasinopoulos et al., 2017). There are other GAMLSS R packages for extra additive terms (gamlss.add), fitting censored (interval) responses (gamlss.cens), fitting finite mixture distributions (gamlss.mx), fitting nonlinear models (gamlss.nl), fitting truncated distributions (gamlss.tr), among others. Other R packages related to GAMLSS are gamboostLSS (Mayr et al., 2012; Mayr & Hofner, 2018) and BAMLSS (Umlauf et al., 2018), and these allow performing boosting methods for GAMLSS models (suitable for high‐dimensional data; see Thomas et al., 2018).

Another appealing feature of GAMLSS are its flexibility for data modeling through estimation algorithms (Cole & Green, 1992; Rigby & Stasinopoulos, 1996) that allow combining ML with statistical modeling (Breiman, 2001b; Stasinopoulos et al., 2018). For example, such algorithms enable fitting the conditional parametric distribution of the response variable with several continuous, discrete, and mixed distributions with different degrees of asymmetry and kurtosis. Therefore, not only the mean, but all of the parameters (i.e. location, scale and shape) can be modeled as parametric and/or additive nonparametric functions of covariates. This feature is quite instrumental to modeling response variables that do not follow an exponential family distribution (some exponential distributions are the Normal, Poisson, Gamma, Beta, Weibull [for fixed shape parameter] and Multinomial distributions) (Barndorff‐Nielsen, 1980; Casella & Berger, 2002; McCulloch, 2000). A study by Voudouris et al. (2012) exemplifies the benefits of finding the right distribution for a data set. Film box‐office revenue data exhibit a positive skew with a heavy tail and it was traditionally modeled via the Pareto–Levy–Mandelbrot (PLM) distribution (a distribution with infinite variance). However, the PLM distribution could not account for the dispersion, skewness, and kurtosis found in the film revenues data and this was impeding making any stable predictions. Voudouris et al. (2012) demonstrated that the four‐parameter Box–Cox power exponential distribution could better fit the data and it allowed correctly predicting, among other things, the price of future contracts indexed by the film's performance. This study thus demonstrates that using a distribution that fits the data well, enables making reliable probabilistic statements. The mechanics of GAMLSS are described next.

Consider a data set (**X**_*k*_, **Z**_*k*_, **y**)_*k*≤*p*_ of sample size *n*, where $y={\left(y_{1},y_{2},\ldots ,y_{n}\right)}^{⊤}$ is a vector of independent observations on the response variable and **X**_*k*_, **Z**_*k*_ are input covariates design matrices for fixed and random effects (a.k.a. features in ML jargon) of size $n\times J_{k}^{\prime }$ and *n* × *q*_*jk*_, respectively. By assuming that the variable of interest follows the probability density function (PDF) $f(y_{i}|\theta^{i}\in D)$, a parametric family of distributions (see table 1 in Rigby & Stasinopoulos, 2005) with ***θ*** ^*i*^ = (*θ*_*i*1_, *θ*_*i*2_, …, *θ*_*ip*_)^⊤^ being a vector of *p* parameters associated to the explanatory variables and to random effects,^3^ each distribution parameter of the GAMLSS model can be written as a function of regressors.

In GAMLSS statistical models, the *k*th parameter ***θ***_*k*_ is related to an additive predictor *η*_*k*_ through input features and random effects via

(1) $$g_{k}\left(\theta_{k}\right)=\eta_{k}=X_{k}\beta_{k}+\sum_{j=1}^{J_{k}}Z_{\mathrm{jk}}\gamma_{\mathrm{jk}},$$

where *g*_*k*_(·) is a strictly monotonic link function, ***θ***_*k*_ = (*θ*_1*k*_, *θ*_2*k*_, …, *θ*_*nk*_)^⊤^ and ***η***_*k*_ = (*η*_1*k*_, *η*_2*k*_, …, *η*_*nk*_)^⊤^ are *n* × 1 vectors, $\beta_{k}={\left(\beta_{1k},\beta_{2k},\ldots ,\beta_{J_{k}^{\prime }k}\right)}^{⊤}$ has dimension $J_{k}^{\prime }\times 1.$ The random effects parameter vector ***γ***_*jk*_ with length $J_{k}^{\prime }$ follows the multivariate Gaussian distribution $N_{q_{\mathrm{jk}}}\left(0,G_{\mathrm{jk}}^{-1}\right),$ where $G_{\mathrm{jk}}^{-1}$ is the inverse of a symmetrical matrix *G*_*jk*_ = *G*_*jk*_(*λ*_*jk*_) of size *q*_*jk*_ × *q*_*jk*_ which depends on a *λ*_*jk*_ hyperparameter vector. If *G*_*jk*_ is singular, then *γ*_*jk*_ has a density function proportional to exp−12γjk⊤Gjkγjk. A GAMLSS model can be expressed differently by including ML procedures in order to boost its predictive power. For example, if *Z*_*jk*_ = *I*_*n*_, where *I*_*n*_ is the identity matrix of type *n* × *n*, and *γ*_*jk*_ = *h*_*jk*_ = *h*_*jk*_(*x*_*jk*_) for all combinations of *j* and *k* expressed in Equation (1), then the GAMLSS model adopts a semi‐parametric additive term:

(2) $$g_{k}\left(\theta_{k}\right)=\eta_{k}=X_{k}\beta_{k}+\sum_{j=1}^{J_{k}}h_{\mathrm{jk}}\left(x_{\mathrm{jk}}\right),$$

where function *h*_*jk*_ is an unknown function of the independent variable *x*_*jk*_ and *h*_*jk*_(*x*_*jk*_) is a vector that evaluates function *h*_*jk*_ in *x*_*jk*_. Furthermore, smoothers such as cubic splines, penalized splines, fractional polynomials, LOESS curves, terms of variable coefficients, neural networks, kernels, and so on, can be included to deal with nonlinearity, volatility structural changes and other particularities in the data (Wood, 2017; Wood et al., 2016).

The vector of fixed and/or random‐effect parameters are estimated within the GAMLSS framework by maximizing the penalized log‐likelihood and this can be accomplished by using fast backfitting algorithms and resampling procedures (Groll et al., 2019; Mayr et al., 2012; Rigby & Stasinopoulos, 2005). Model selection is performed by finding the lowest generalized Akaike information criterion [GAIC(*k*)] for some selected value of *k* in the same context of AIC (Akaike, 1974) along with cross‐validation (Geisser, 1975; Voncken et al., 2019) in order to prevent over‐fitting of the data. The GAIC is defined by Voudouris et al. (2012) as GAIC(*k*) = GD + (*k* × *g*_*l*_), where $\mathrm{GD}=-2\ell \left(\hat{\theta }\right)$ is the global deviance being $\ell \left(\hat{\theta }\right)$ the maximized log‐likelihood function, *g*_*l*_ denotes the total effective degrees of freedom of the adjusted model and *k* is a constant penalty for each degree of freedom used. If *k* = 2, the GAIC equates to the AIC, and if k=lnn it equates to the Bayesian information criterion (BIC). For the analysis of residuals, the normalized (randomized) quantile residuals plot can be used (Dunn & Smyth, 1996). In addition, GAMLSS allow examining residuals via probability plots such as the worm plot (van Buuren & Fredriks, 2001) which is an instrumental graphical technique for assessing the overall adequacy of the fitted model (see Fasiolo et al., 2020; Stasinopoulos et al., 2017).

By using family of sets notation, a GAMLSS model can be represented for ML implementation as

(3) $$ℳ=\left\{D,G,T,\lambda \right\},$$

where D represents a family of distributions, G specifies the set of link functions $\left(g_{1},\ldots ,g_{p}\right)$ for parameters $\left(\theta_{1},\ldots ,\theta_{p}\right),$ T specifies the set of predictor terms $\left(\eta_{1},\ldots ,\eta_{p}\right)$, and *λ* specifies the set of hyperparameters. Thus, the linear regression model, for example, can be written as $y_{i}\sim D\left(g_{1}\left(\mu \left(x\right)\right),g_{2}\left(\sigma {\left(x\right)}^{2}\right)\right)$, where D is the normal distribution, $G=\left\{g_{1},g_{2}\right\}=\left\{\mathrm{id}\left(x\right),\mathrm{id}\left(x\right)\right\}$, and *λ* = (*β*, *σ* ^2^), where id(*x*) is the identity function. Note that in this case, *μ*(*x*) = *x* ^⊤^ *β* and *σ*(*x*)^2^ = *σ* ^2^. Another important example is logistic regression or softmax regression in the context of neural networks. In such case, a GAMLSS model can be expressed as $y_{i}\sim D(g_{1}\left(p\left(x\right)\right)),$ where D is the Bernoulli distribution, $G=\left\{g_{1}\right\}=\left\{\text{logit}\left(x\right)=\log\left(x/\left(1-x\right)\right)\right\}$ (logistic or softmax function), and $p\left(x\right)=P\left(y_{i}=1|x_{i}\right)=\exp\left(x_{i}^{⊤}\beta \right)/\left(1+\exp\left(x_{i}^{⊤}\beta \right)\right)$ with *x* ^⊤^ *β* being a linear predictor.

In order to compare two nested competing GAMLSS models ${ℳ}_{0}$ and ${ℳ}_{1}$ based on Equation (3), that is, when one model can be obtained from the others by imposing parametric restrictions, the global deviance or a LASSO approach (Groll et al., 2019) can be used to penalize overfittings and select the best model. When comparing two non‐nested GAMLSS models (including models with smoothing terms; Hastie & Tibshirani, 1990), the GAIC (Akaike, 1974) and the *J* and *MJ* tests can be used (Cribari‐Neto & Lucena, 2017; Davidson & MacKinnon, 1981; Godfrey, 2011; McAleer, 1995).

In a nutshell, GAMLSS are a framework that uses state‐of‐the‐art algorithms for the modeling of continuous responses. As shown above, distributional regression analyses within a GAMLSS framework permit smooth alignment with well‐known methods in ML. Although there are methods to compare data's means and standard deviations (Frank & Klar, 2016) and data's kurtosis and skewness (Cain et al., 2017), GAMLSS are a unified framework that promotes going beyond traditional mean regression (Kneib, 2013; Kneib et al., 2021) by considering other moments of the dependent variable's distribution. The GAMLSS framework is thus in line with recent proposals of moving beyond means and standard deviations to, at minimum, data's location and scale (Trafimow et al., 2018).^4^ A final aspect to reiterate is that GAMLSS are designed to be a flexible and interpretable regression‐based method for statistical learning. This is a beneficial feature to counter “black‐box” modeling and instead facilitate models' explainability and applicability (see Yu & Kumbier, 2020). For more details on GAMLSS, see Stasinopoulos et al. (2017) and Rigby et al. (2020).

## 3 THE OPEN UNIVERSITY LEARNING DATA SET

The goal of this article is to overview some of the statistical modeling capabilities of GAMLSS through a data set from the field of LA/EDM. The Open University Learning Data Set (OULAD; Kuzilek et al., 2017) is an open‐access data set of about 32,593 students in a distance learning setting that relies on a virtual learning environment (VLE; for other data sets in the LA/EDM field see Mihaescu & Popescu, 2021). This data set contains a diverse set of students' attributes obtained from a large sample of university students. The OULAD is thus a suitable data set for testing new approaches to the predictive modeling of students' outcomes, their behaviors in VLEs, and evaluation of new approaches to LA. For example, Alshabandar et al. (2018) used Gaussian mixture models for the prediction of passing the next assessment based on clickstream data. Other models such as *k*‐nearest neighbors, Naive Bayes, Decision Trees, Random Forests (RF), or support vector machines (SVM) have also been used for predicting the results of studied courses (Azizah et al., 2018; Ho & Jin Shim, 2018; Rizvi et al., 2019; Silveira et al., 2019). Finally, the OULAD has also been used for the evaluation of deep learning approaches for estimating students' withdrawal (Hassan et al., 2019), for distinguishing groups of students based on their activities in VLEs via unsupervised learning methods (Heuer & Breiter, 2018; Peach et al., 2019), and for the evaluation of methods of course recommenders based on the detection of learning styles (Li et al., 2019).

The OULAD has been released by the Open University; the largest distance learning institution in the United Kingdom with more than 165,000 students and hundreds of courses. Regular courses take approximately 9 months to study and consist of multiple assignments and a final exam. The assignments can be divided into various categories being the Tutor Marked Assignments (TMAs) the most important as it represents key milestones in a study's schedule. The university employs a Moodle‐like online system to deliver the course content to the students. This allows capturing valuable information such as students' demographics, study results, and their behavior within the VLE represented by the summaries of click‐stream data.

**FIGURE 1:**
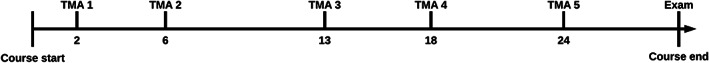
Course schedule (timeline occurs in weeks).

One particular STEM (science, technology, engineering, and mathematics) course has been selected for the present analysis; FFF and its presentation (semester) *2013J* studied by 2283 students. The course schedule is represented in Figure 1. The course contains five TMAs that represent the milestones for the topic learning periods. TMAs occur in weeks 2, 6, 13, 18, and 24 and at the end of the course an exam is taken. The exam takes place around four or more weeks have passed (in the current data set such information is missing). The present GAMLSS analysis focuses on this last TMA (TMA 5) (in the data set it is labeled assessment_score). TMA 5 is thus the dependent variable and it ranges between 0 and 100, such that values over 40 are considered as pass.

The following groups of students were excluded from the data set: actively withdrawn students (*n* = 675) and students who did not submit all TMAs (*n* = 500). Actively withdrawn students were unregistered from the course before its end, and their information regarding VLE activities and assessments is incomplete. The second group did not submit all the assignments in time as required by the course. The resulting data set thus contains data of 1108 students. Table 1 lists and describes the independent variables in the data set. The first column contains the name of the variables and the second column shows a brief description of each variable (more details as to the source data set can be found in Kuzilek et al., 2017). The click‐stream information (i.e., “clicks_xy” variables) has been computed for the top five most common activity types in the VLE, and they represent 95% of all student click‐stream data.

**TABLE 1:** List of independent variables

Attribute	Description
Gender	Student gender
Region	UK region, in which student lives^a^
Highest_education	The highest achieved education of the student
Imd_band	Percentile of the Index of Multiple Deprivation; see Noble et al. (2019) for details
Age_band	Student age band
Num_of_prev_attempts	Indicator whether the student attempted the course in previous years
Studied_credits	Credits studied in parallel by student, serves as the estimation of student workload
Disability	Indicator if student have disability
Cumulative_assessment_results	Weighted sum of all previous Tutor Marked Assignments (TMAs): $a_{\mathrm{sum}}=\sum_{n=1}^{4}w_{n}a_{n},$ where ${\vec{w}}^{T}=\left(0.125,0.125,0.25,0.25\right)$ is vector of corresponding weights
Clicks_forumng	Sum of all clicks/actions student did in the discussion forum
Clicks_homepage	Sum of all clicks on course homepage
Clicks_oucontent	Sum of all views/clicks on TMAs assignments
Clicks_quiz	Sum of all clicks/attempts on nongraded quizzes
Clicks_subpage	Sum of all clicks when browsing the course web‐page

^a^ The complete list of regions can be found at https://bit.ly/3kKF1zs.

## 4 STATISTICAL LEARNING OF THE OULAD DATA SET VIA GAMLSS

The goal of the following modeling is to illustrate how GAMLSS can be used in practice and has no attempt at making theoretical LA/EDM‐related claims based on the OULAD data set. The first step in GAMLSS modeling is to find a set of suitable marginal distributions (i.e., when the dependent variable is not conditioned on any covariates) that approximate well the observed values. The dependent variable was linearly transformed so that its values resided in the [0, 1] interval; that is, FAS = assessment/score/100; where 100 is the maximum assessment score (GAMLSS modeling of these types of data can be found in Ospina & Ferrari, 2012a, 2012b).

GAMLSS enable to fit several distributions to the target variable via the histDist() and fitDist() functions and in this study only the latter was used. Given that the marginal distribution is left‐skewed and bounded in the [0, 1] interval, the extra arguments type = "real0to1" or type = "realline" in fitDist() can be used to exhaustively search for suitable distributions. The output of the search returns global deviance, AIC, and BIC values that assist in spotting candidate distributions. To avoid numerical problems, zeros and ones were converted to 0.5/100 and to 99.5/100, respectively (see Douma & Weedon, 2019). Note that choosing the distribution, or a set of candidate distributions, is not only a matter of statistical fitness but also of practical interpretability. Distributions with three or more parameters will tend to fit the skewness and kurtosis of the distribution better than distributions with one (e.g., Exponential) or two (e.g., Gamma) parameters. However, the applied researcher should prioritize distributions that parsimoniously explain changes in the values of the dependent variable in relation to the covariates in the context of the topic of the research. Table 2 shows the AIC measures of several distributions fitted to the marginal FAS distribution (GAMLSS have over 100 distributions available in the gamlss.dist package, loaded by default with the gamlss package).

**TABLE 2:** Akaike information criterion (AIC) and global deviance (GD) for the fitted models with different probability distributions. The lower the AIC value, the better the goodness‐of‐fit. For example, generalized Beta type 1 (GB1) to Skew *t*‐distribution (SST) are four‐parameter distributions, while Logistic (LO) to Reverse Gumbel (RG) are two‐parameter distributions (except the exGAUS, Ex‐Gaussian distribution, which is a three‐parameter distribution).

Distribution	AIC	GD
**GB1**	−5593	−5601
ST2	−5556	−5564
ST3	−5556	−5564
ST1	−5556	−5564
**SST**	−5544	−5564
SN2	−5438	−5564
EGB2	−5395	−5564
SHASH	−5378	−5564
SEP3	−5375	−5564
JSUo	−5362	−5564
JSU	−5362	−5564
SHASHo2	−5361	−5564
SHASHo	−5361	−5564
ST5	−5234	−5564
LOGITNO	−5224	−5601
BE	−5080	−5601
BEo	−5080	−5601
BEOI	−5078	−5601
BEZI	−5078	−5601
BEINF1	−5078	−5601
BEINF0	−5078	−5601
BEINF	−5076	−5601
GU	−4733	−5564
ST4	−4319	−5564
GT	−3474	−5564
NET	−3367	−5564
TF2	−3360	−5564
TF	−3360	−5564
PE	−3218	−5564
PE2	−3218	−5564
**LO**	−3178	−5564
NO	−2732	−5564
**exGAUS**	−2729	−5564
SIMPLEX	−2392	−5601
**RG**	168	−5564

**FIGURE 2:**
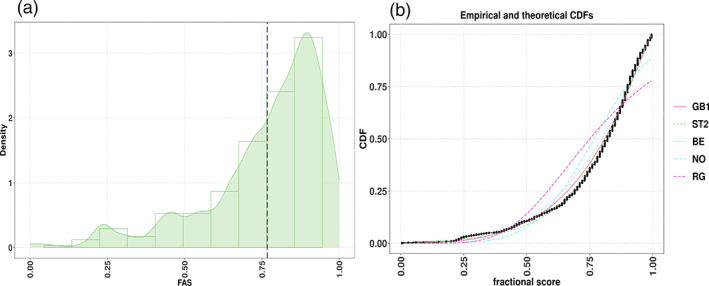
FAS′ kernel density estimates superimposed on histogram (a) and FAS′ empirical and theoretical CDFs (b). The vertical dotted line in the left plot indicates the variable's mean. The black line in the right plot shows the FAS′ ECDF and the colored lines represent five theoretical CDFs (ranked from best GB1 to worst fit RG). CDF, cumulative distribution functions; ECDF, empirical CDF; GB1, generalized beta type 1; RG, reverse Gumbel.

The PDF and empirical cumulative distribution function (ECDF) plots indicate a negative skewness in FAS (see Figure 2a). It is evident from the CDF plot that the Normal (NO) and Reverse Gumbel (RG) distributions provide poor marginal fits even though these distributions are encountered in practical work (Rigby et al., 2020). The Beta class distributions (BE, BEINF, etc.) are natural candidates (Ospina & Ferrari, 2010) and exhibit reasonable behavior. On the other hand, the generalized beta type 1 (GB1) and Skew *t*‐type 2 distributions (ST2) (Azzalini & Capitanio, 2003; Rigby et al., 2020) gave the best fits.

**FIGURE 3:**
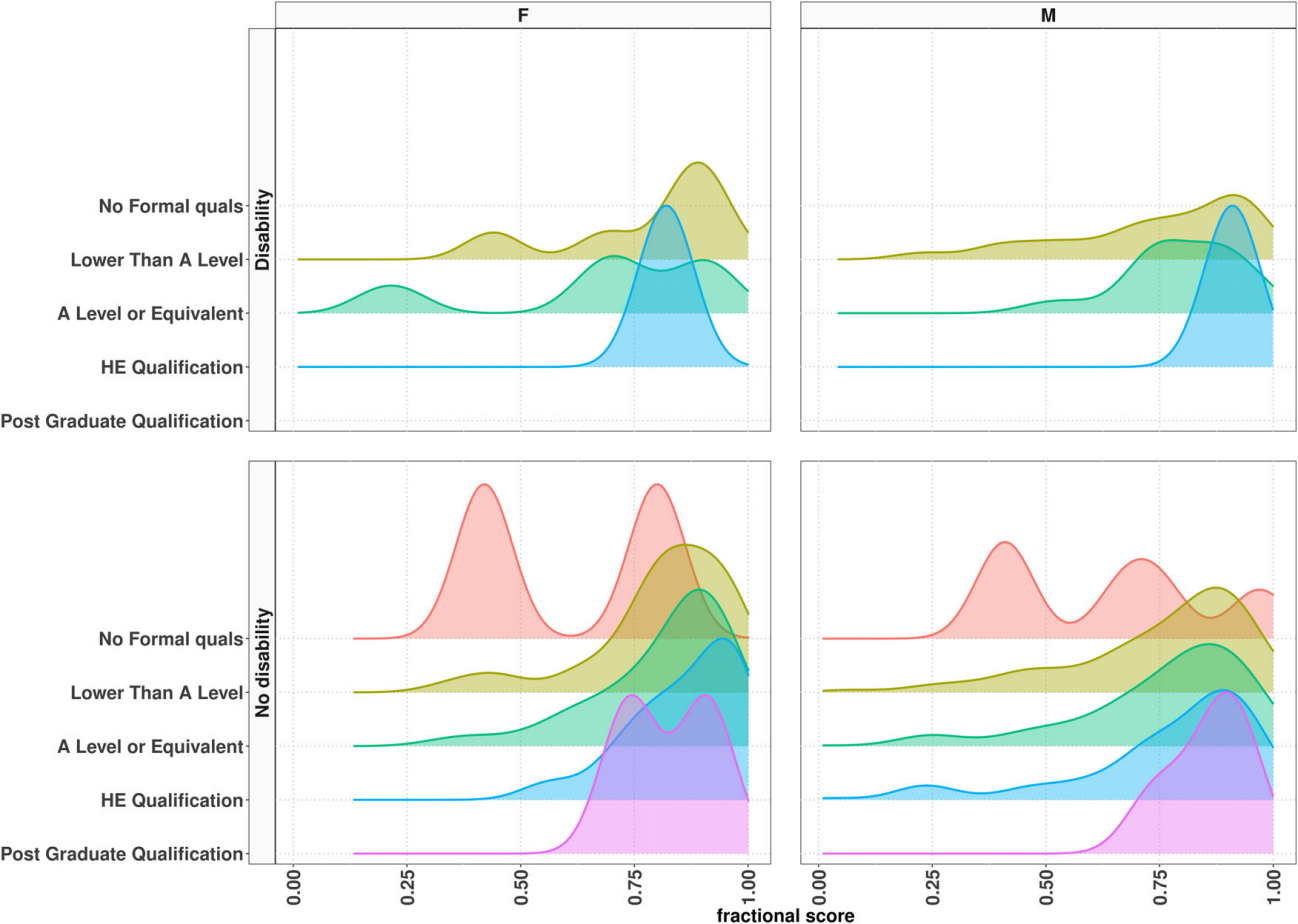
FAS′ kernel density estimates conditioned on the covariates gender (with two levels; F = females and M = males), disability (with two levels; first row = disability, second row = no disability) and highest education (with five levels). The graph also indicates the data are imbalanced in that not all combinations of levels of the covariates have values. That is, while there are FAS values for people with nondisability at all education levels, there are FAS values for people with disabilities at three education levels only.

Figure 3 shows FAS' kernel density estimates conditioned on the covariates gender, disability, and highest education. These PDF plots are exploratory data analysis (EDA) tools (Tukey, 1977) that allow noticing differences in the location, dispersion and shape of the conditional distribution of FAS (i.e., for each combination of covariates). For example, it is evident that the higher the educational qualification, the higher the FAS and that students with disability tend to have lower FASs than nondisability students. Therefore, a useful approach to analyze the relation between FAS and its covariates is via GAMLSS as it allows to learn changes in the location, and other parameters, of the distribution of FAS as influenced by its covariates.

Using Wilkinson and Rogers' notation (Wilkinson & Rogers, 1973), the model to consider is FAS ∼ *C* + *N*, where FAS is the dependent variable (fractional assessment_score), *C* represents a matrix with categorical covariates (gender, highest_education, age_band, disability, and type_of_click), and *N* is a numeric covariate (number of clicks). For illustration purposes, parametric fixed‐effects‐only regression models are specified, the set of distributions {NO, BE, ST2, GB1} is the response distribution space for the search and the scale and shape parameters are assumed to be constant for all observations; that is, the focus is on the *μ* location parameter only.^5^

More specifically, the conditional models considered here have the same linear predictor:

(4) η=gender+age_band+disability+highest_education+type_of_click+number_of_clicks.

The *μ* submodel has the following link functions: *g*_NO_(*μ*) = identiy(*μ*) ∼ *η*, *g*_BE_(*μ*) = logit(*μ*) ∼ *η*, *g*_ST2_(*μ*) = identiy(*μ*) ∼ *η*, and *g*_GB1_(*μ*) = logit(*μ*) ∼ *η*, respectively. Note that the Normal distribution (i.e., NO) was included for illustration purposes. Variable selection for the location submodel was made via the stepGAIC method in GAMLSS (Stasinopoulos et al., 2018) (note the drop1() function is also useful for this goal). The selection of the distribution was based on the examination of AIC and quantile residuals via worm plots.

Table 3 presents the comparison of the fitted models with different distributions. As expected, the Normal distribution gave a poor fit (see also Figure 2). On the other hand, and as shown by the ECDF plots (see Figure 2b), the GB1 and ST2 distributions showed the best performance as indexed by the Likelihood, AIC and degrees of freedom estimates.^6^ The predictive power of the selected model was assessed via the general pseudo‐$R_{C\&S}^{2}$ (Cox & Snell, 1968; Nagelkerke, 1991) (implemented in the GAMLSS R function Rsq()) and the pseudo‐$R_{S}^{2}$ (which is given by the square root of Spearman's sample correlation coefficient between the response and the fitted values; this approach is valid only for location submodels). The pseudo‐$R_{S}^{2}$ measures suggest that a model using the GB1 distribution gives the highest predictive power.

**TABLE 3:** Goodness‐of‐fit measures of selected *μ* submodels

*μ* submodel	Distribution	Likelihood	AIC	Degrees of freedom	Pseudo‐$R_{C\&S}^{2}$	Pseudo‐$R_{S}^{2}$
*g*_GB1_(*μ*)	GB1	−5629	−5597	5194	0.072	0.126
*g*_ST2_(*μ*)	ST2	−5282	−5250	5194	0.021	0.122
*g*_BE_(*μ*)	BE	−5239	−5211	5196	0.089	0.125
*g*_NO_(*μ*)	NO	−3087	−3087	5196	0.083	0.122

Abbreviations: AIC, Akaike information criterion; BE, Beta class distribution; GB1, generalized Beta type 1; NO, Normal distribution; ST2, Skew *t*‐type 2 distribution.

**FIGURE 4:**
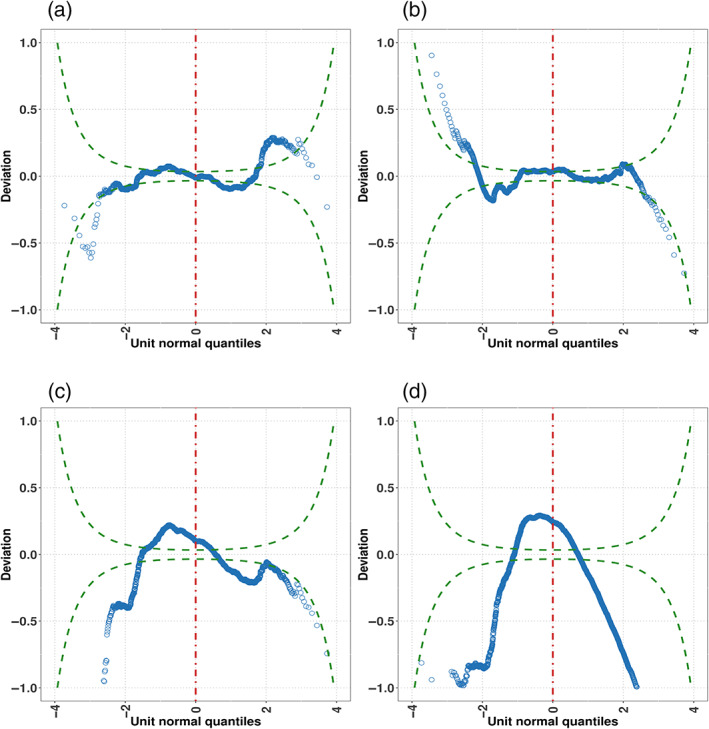
Diagnostic worm plots for assessing the fitness of models using the generalized beta type 1 (GB1) distribution (a), Skew *t*‐distribution type 2 (ST2) (b), Beta (BE) distribution (c), and Normal (NO) distribution (d) to the FAS variable. A good fit is represented by ≈ 95% of values lying between the two green dotted elliptic lines and close to the deviation value of 0.0. In this example, the GB1 and ST2 distributions fit well most of the data but they struggle to fit the values in the tails of the FAS variable (although the ST2 distribution models better the right tail of the data than the GB1 distribution). However, compared to the GB1 and ST2 models, BE and NO exhibit a poor fit overall.

Figure 4 shows the gamlss fit output of the GB1 *μ* model^7^ (this output was obtained via the generic function summary()). After a submodel selection step, the resulting *μ* submodel was:

(5) $$\text{logit}\left(\mu \right)\sim \text{gender}+\mathrm{age}\_\text{band}+\text{highest}\_\text{education}+\text{type}\_\text{of}\_\text{click}+\text{number}\_\text{of}\_\text{clicks}.$$

The results indicate that all the covariates, have effects on the *μ* parameter of the response variable FAS.^8^ There was no evidence for an effect of “disability” possibly due to this variable presenting unbalanced information as shown Table 4, such situation could be addressed by obtaining more data or using bootstrap to obtain more confidence in the generality of the model (Branco et al., 2018).

On the other hand, provided all other variables are held constant, an increase in age after 55 is related to a decrease in FAS' location. Likewise, all other variables fixed, an increase in the number of clicks increases the location of the FAS distribution.

The adequacy of the fitted distributions is represented in Figure 4 via worm plots. A lack of fit is displayed by the residuals lying well above and below the value deviation 0.0. Also, the less closer the points of the plot are to the horizontal line at 0.0, the more distant the distribution of the residuals is to a standard normal distribution. In addition, a lack of fit is suggested when more than 5% of the points of the plot lie outside the two elliptic lines (those elliptic lines are point‐wise ≈ 95% confidence intervals [CIs]). The results of the Beta, but particularly the Normal, distribution showed lack of fit and their inverted U‐shape also signaled negative skewness in the residuals' distribution. This inverted U‐shape also indicated that those distributions failed to correctly fit the high left‐skewness of the data. Although the worm plot shapes of the GB1 and ST2 distributions suggested good fit, it was not perfect. Both struggled to fit the kurtosis of the marginal distribution and this is evidenced in the distribution of the residuals being leptokurtic in the case of the GB1 distribution (S‐shape with left bent down) and platykurtic in the case of the ST2 distribution (S‐shape with left bent up). Also, that some of the points in the plots representing the GB1 and ST2 distributions laid outside the ≈ 95% CIs indexes some degree of overdispersion in the data (see chapter 12 in Stasinopoulos et al., 2017 for details as to the interpretation of the worm plot).

The modeling performed here was fully parametric; so smoothers are to be used if a semi‐parametric modeling is sought. In this sense, GAMLSS allow adding nonparametric smoothing functions for numeric covariates in order to augment the prediction power. Some of the functions available are: cubic splines, decision trees, locally weighted regression, penalized splines, and neural networks (Rügamer et al., 2020). Generally, it is recommended to use P(enalized)‐splines (Eilers & Marx, 1996) in order to include potentially local and nonlinear effects of continuous variables (Wood, 2017; Wood et al., 2016). As illustration, the location parameter *μ* of the GB1 distribution was modeled using a penalized P‐spline function as a way of understanding how the dependent variable FAS is affected by the covariates. Specifically, the nonparametric smoother pb() in gamlss was applied to the covariate number_of_clicks to capture local variations in the context of the following model:

(6) $$\eta =\text{gender}+\mathrm{age}\_\text{band}+\text{disability}+\text{highest}\_\text{education}+\text{type}\_\text{of}\_\text{click}+\mathrm{pb}\left(\text{number}\_\text{of}\_\text{clicks}\right).$$

**FIGURE 5:**
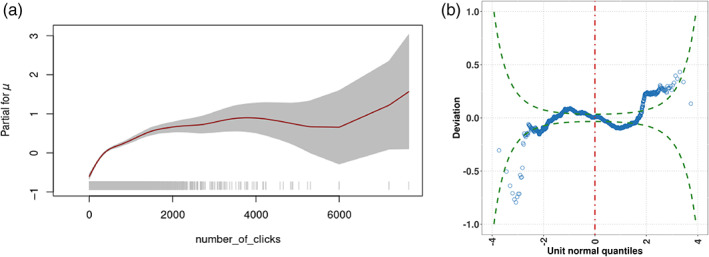
Termplot for the *μ* submodel when it includes a smooth term (P‐splines) on the covariate “number of clicks” (a). Plot (b) shows the diagnostic worm plot for assessing the fitness of the GB1 model.

To facilitate the interpretation of these predictors, the function term.plot() in the gamlss package was used. This function produces plots of parameter estimates (in the link function scale) for each covariate in the predictor of each parameter of the population distribution. Point estimates are represented by the trend lines (linear or smooth predictor) in Figure 5a and the shaded areas correspond to the estimates' standard errors. The plot suggests an increasing nonlinear relationship between FAS and the number of clicks. However, the relationship is not monotonic and the change in standard error suggests heteroskedasticity likely due to the presence of groups or clusters (indeed, data sparsity at the upper end of the covariate spectrum could also have played part in this effect). As it was the case of the fully parametric GB1 model, the *μ* term was not affected by “disability” after the submodel selection procedure. The inclusion of the nonparametric term increased the performance of the AIC (−5917) and the predictive power; pseudo‐$R_{C\&S}^{2}$ (0.131) and pseudo‐$R_{S}^{2}$ (0.14). The worm plot in Figure 5b suggests that the inclusion of the nonparametric term helped to control the GB1's right tail (compare Figure 4a vs Figure 5b) (see Supporting Information for a deeper discussion of the estimated effect of covariates on the conditional response distribution).

So far, all the GAMLSS modeling has been done only on the location parameter (*μ*) of the dependent distribution. A way to boost predictive power is by also modeling the other parameters of the dependent variable. A likelihood ratio aimed at determining whether the GAMLSS scale and shape parameters were constant for all observations suggested these parameters were not constant. Thus, the linear predictor given in Equation (4) was applied to the GB1 distribution's parameters through their *σ*, *ν*, and *τ* link functions; that is, log(*σ*) ∼ *η*, log(*ν*) ∼ *η*, and log(*τ*) ∼ *η*, respectively. As done above for the FAS' location parameter *μ*, recursive covariate selection based on AIC was performed for the scale and shape parameters. The results of this selection procedure showed that all submodels were distinctively affected by the covariates such that,

$$\begin{array}{l} \text{logit}\left(\mu \right)\sim \text{gender}+\mathrm{age}\_\text{band}+\text{type}\_\text{of}\_\text{click}+\text{number}\_\text{of}\_\text{clicks}\\ \log\left(\sigma \right)\sim \mathrm{age}\_\text{band}+\text{highest}\_\text{education}\\ \text{logit}\left(\nu \right)\sim \mathrm{age}\_\text{band}+\text{number}\_\text{of}\_\text{clicks}\\ \log\left(\nu \right)\sim \text{highest}\_\text{education}. \end{array}$$

**FIGURE 6:**
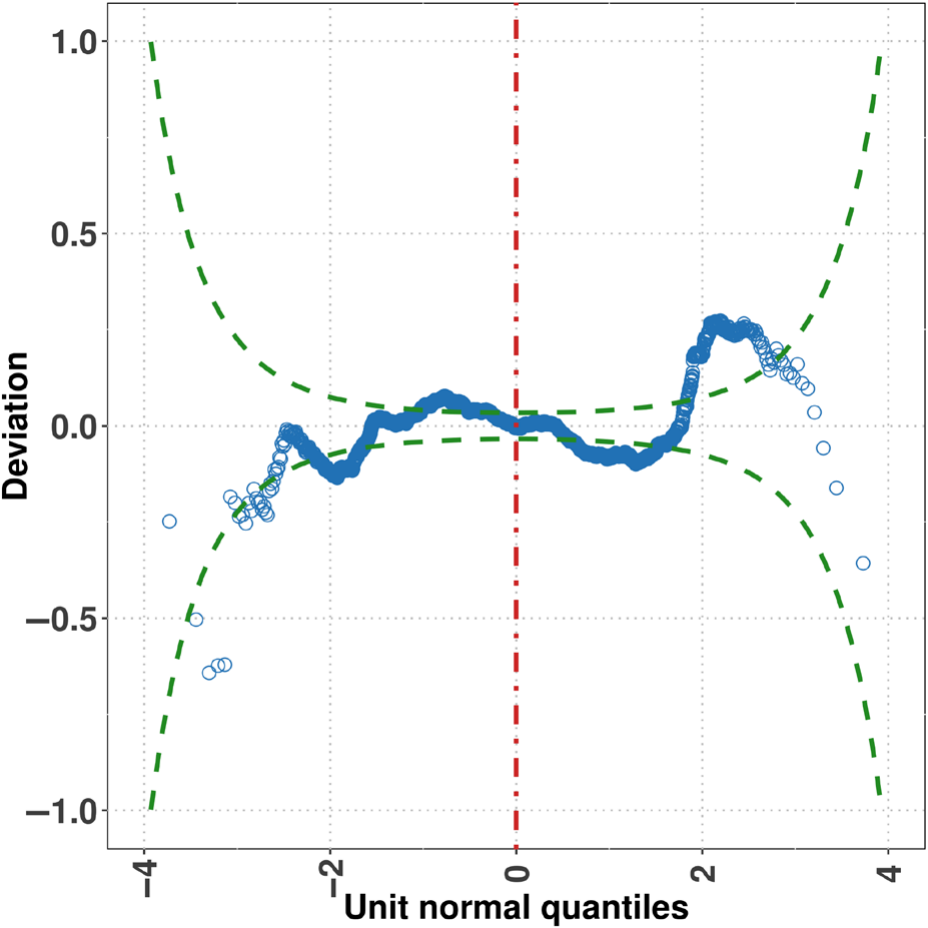
Worm plot for the GB1 model when the *μ*, *σ*, *ν*, and *τ* parameters were modeled.

This new model showed a pseudo‐$R_{C\&S}^{2}$ of 0.12. That is, there was a 15% increase in performance improvement when compared with the model obtained in Equation (5) (model without smoothers). Note also that after the submodel selection procedure, the ‘disability’ covariate was not part of the predictors once again. Figure 6 displays the residual worm plot for this comprehensive model. This new model indicated that fitting all the parameters of the FAS' distribution led to minimizing the leptokurtosis in the residuals; that is, the points in the left tail of the worm plot are now closer to the ≈ 95% boundaries (compare Figures 4a vs. 6).

GAMLSS allow using complementary techniques to improve the modeling of the data but it would be prohibitive to attempt to cover them all herein. Thus, some techniques are briefly commented on. Variable selection can be carried out via cross‐validation or LASSO in order to control over‐fitting by considering different link functions for the covariates (e.g., identity, inverse, reciprocal, etc.). An example of this practice can be found in Cribari‐Neto and Lucena (2017). Also, the number of levels in categorical covariates can be reduced in order to improve the fitness of the model (see pcat() function in GAMLSS). GAMLSS also permit to robustify the model's fitness by countering the influence of outliers (via the function gamlssRobust).

**TABLE 4:** Summary results of the generalized Beta type 1 distribution (GB1) when modeling the *μ* (location) submodel

Family: c("GB1," "Generalized Beta type 1") Call: gamlss(formula = response ~ gender + age_band + disability + highest_education + type_of_click + number_of_clicks,family = GB1, data = na.omit(data)) Fitting method: RS() ––––––––––––––––––––––––––––––––– Mu link function: logit Mu Coefficients:

Variable	Estimate	SE	*t*‐value	Pr(>|*t*|)
Intercept	0.8790	0.1437	6.1153	0.0000***
genderm	−0.2211	0.0321	−6.8800	0.0000***
Age_band35‐55	0.1283	0.0287	4.4637	0.0000***
Age_band55<=	−0.7917	0.1518	−5.2153	0.0000***
Disability_No.disability	0.0496	0.0460	1.0792	0.2805
Highest_educationHE.Qualification	0.0790	0.0352	2.2438	0.0249*
Highest_educationLower.Than.A.Level	−0.0744	0.0260	−2.8627	0.0042**
Highest_educationNo.Formal.quals	−0.9865	0.1537	−6.4180	0.0000***
Highest_educationPost.Graduate.Qualification	0.0444	0.1794	0.2476	0.8045
Type_of_clickclicks_homepage	0.0125	0.0331	0.3780	0.7054
Type_of_clickclicks_oucontent	−0.2863	0.0385	−7.4392	0.0000***
Type_of_clickclicks_quiz	−0.0051	0.0285	−0.1770	0.8595
Type_of_clickclicks_subpage	0.0886	0.0348	2.5464	0.0109*
Number_of_clicks	0.0005	0.0000	16.2857	0.0000***

Cross‐validation is a ubiquitous step in ML. In GAMLSS, *k*‐fold cross‐validation is attained via the gamlssCV() function. If the goal is to fit a gamlss model to the training data set and estimate the validation global deviance for the validation data set, the gamlssVGD() function can be used. It is important to recall, though, that cross‐validation requires relative stability of the structured data and complete observations for each level within each variable in order to obtain reliable estimates (Gronau & Wagenmakers, 2019; Keevers, 2019; Wang & Gelman, 2015). As shown in Figure 3, some levels within the education level variable had missing observations for males and females and with disability/nondisability; this situation thus led to estimation issues when cross‐validation was attempted. Finally, missing values can be handled by creating predictive models that include imputation or LASSO‐type regularization (Arrieta et al., 2020; Hamzah et al., 2020).

### 4.1 Comparison of GAMLSS to selected ML methods

A study investigating the performance of GAMLSS against one ML method showed that GAMLSS outperformed artificial neural networks in the modeling of war‐fighting combat simulation data (Boutselis & Ringrose, 2013). This section aims at evaluating how GAMLSS perform in relation to other ML algorithms during the modeling of the OULAD data set. Four ML methods were considered:

- **Classification and regression tree (C&RT)** (Breiman et al., 1984): This classic method builds and prunes a decision tree using, for example, Gini's impurity measure. The decision tree itself provides an explanation of each decision and it is simple to understand. However, building such a decision tree is sensitive to the input data, and even small changes in the data can result in a large change in the final model.
- **Random Forest (RF)** (Breiman, 2001a): This is another ensemble learning approach, which uses the ensemble of decision trees for classification and regression.
- **Extreme gradient boosting (EGB)** (Chen & Guestrin, 2016): This is an efficient variant of the ensemble learning proposed by Chen and Guestrin in 2006.
- **Nonlinear support vector machines with radial basis function kernel (nlSVM+k)** (Murphy, 2012): This is an algorithm which constructs the decision boundary based on the structure of the input data. The kernel approach maps data into a higher dimension in order to reduce error caused by nonlinear relationships. This kernel method was used here.

The interest here is not to identify the best model for inference effects (covariate selection) for scientific insight and interpretation. All models with the features used in the linear predictor in Equation (4) were selected and trained in order to make a fair model comparison (Ding et al., 2018; Emmert‐Streib & Dehmer, 2019). A 10‐fold cross‐validation approach was used to validate the models. First, the data were divided into 10 folds and in every step 1 fold was used for the model validation and the rest for the model training. The training set (9 folds) was again divided in 10 folds and cross‐validation was used for tuning the models' parameters. Models were compared via the root mean square error (RMSE), mean absolute error (MAE), and the coefficient of determination (*R* ^2^) metrics^9^:

(7) $$\begin{array}{l} \text{RMSE}=\sqrt{\frac{1}{N}\sum_{i=1}^{N}{\left(y_{i}-\hat{y_{i}}\right)}^{2}},\\ \mathrm{MAE}=\frac{1}{N}\sum_{i=1}^{N}|y_{i}-\hat{y_{i}}|,\\ R^{2}=1-\frac{\sum_{i=1}^{N}{\left(y_{i}-\hat{y_{i}}\right)}^{2}}{\sum_{i=1}^{N}{\left(y_{i}-\bar{y}\right)}^{2}}, \end{array}$$

where *y*_*i*_ represents the assessment score (FAS), $\hat{y_{i}}$ is the predicted FAS, *N* is the data set's sample size, and $\bar{y}$ is the mean value of the FAS in the OULAD.

**FIGURE 7:**
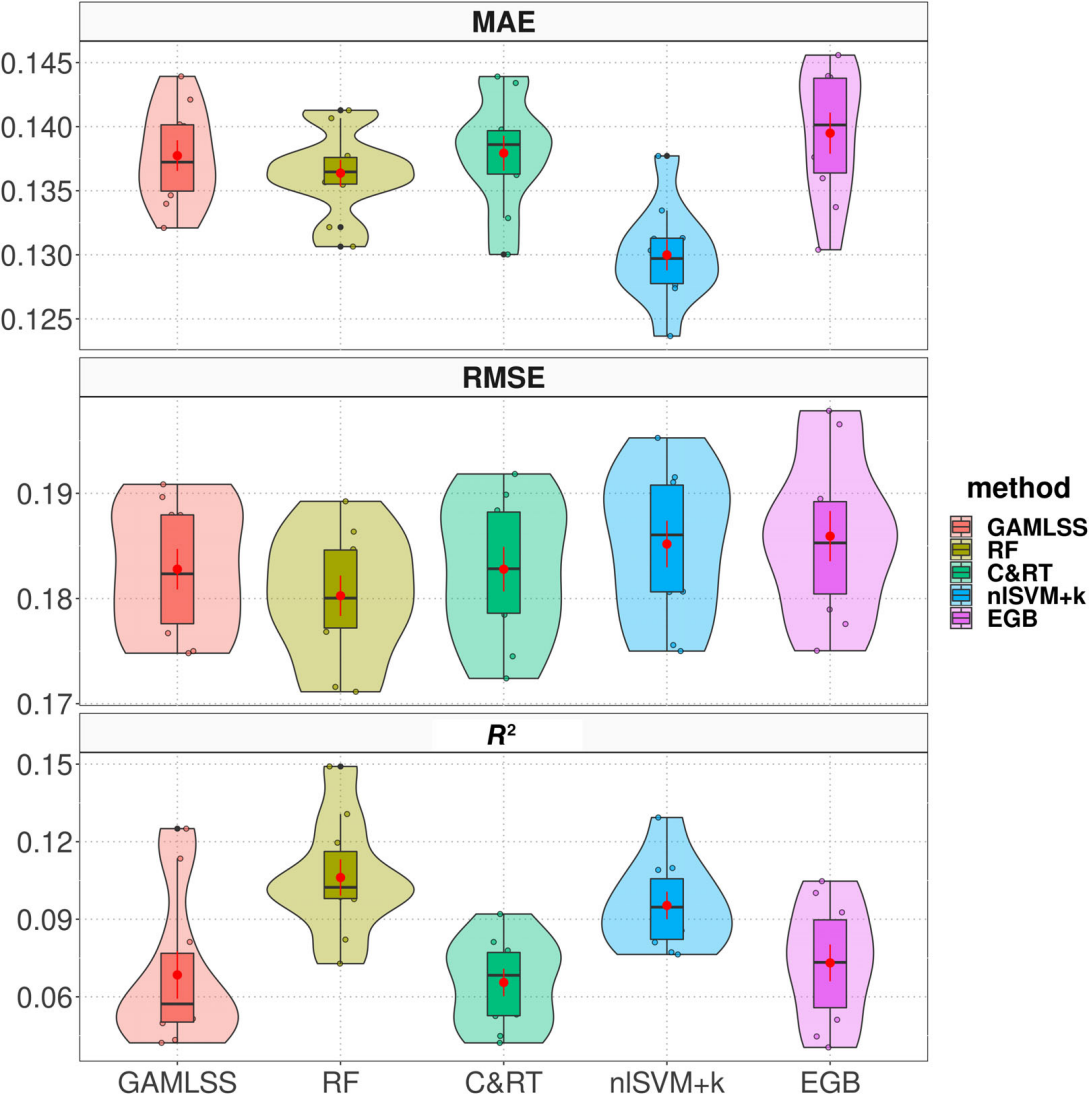
Violinplots of the cross‐validation results. The mean and its 95% confidence interval (CI) are represented by the red dots and error bars. The overlaid dot plots, on each violin plot, represent the result of each of the 10‐fold cross‐validation.

The results of the models' performance are shown in Table 5 and in the Figure 7. Overall, there were minimal differences among the models; that is, all methods showed similar predictive power. Although the RF, C&RT, nlSVM+k, and EGB methods tend to be considered as having relatively high flexibility (see figure 2.7 in James et al., 2017) and accuracy (see figure 12 in Arrieta et al., 2020), they also have low interpretability (see those same figures). GAMLSS, however, given its superset relationship to GLM and GAM, can be regarded as also being flexible and accurate but allowing higher interpretability. In other words, while the RF, C&RT, nlSVM+k, and EGB methods could be regarded as “black‐box” models, GAMLSS can be regarded as a “white‐box” model. Indeed, even if GAMLSS had mid‐range flexibility and accuracy, its higher level of interpretability is in line with what future techniques in ML (including artificial intelligence) are striving for (Angelov et al., 2021; Gunning et al., 2019). Thus, the semi‐parametric GAMLSS model is an educated and interpretable choice to produce insights into the OULAD data set.

**TABLE 5:** Performance of four ML methods and GAMLSS when applied to the OULAD. The best metrics are shown in bold characters (i.e., the lowest RMSE and MAE and the highest *R* ^2^). Means (*M*) and standard deviations (SD) are estimated across 10‐fold cross‐validation.

Method	RMSE		*R* ^2^		MAE	
	*M*	SD	*M*	SD	*M*	SD
GAMLSS	0.1828	0.0061	0.0685	0.0291	0.1377	0.0038
RF	**0.1803**	0.0061	**0.1061**	0.0223	0.1364	0.0033
C&RT	0.1828	0.0067	0.0655	0.0168	0.1379	0.0043
nlSVM+k	0.1852	0.0070	0.0953	0.0168	**0.1300**	0.0038
EGB	0.1859	0.0075	0.0731	0.0225	0.1395	0.0051

Abbreviations: C&RT, classification and regression tree; EGB, extreme gradient boosting; GAMLSS, generalized additive models for location, scale, and shape; MAE, mean absolute error; ML, machine learning; nlSVM+k, nonlinear support vector machines with radial basis function kernel; OULAD, Open University Learning Dataset; RF, Random Forests; RMSE, root mean square error.

## 5 DISTRIBUTIONAL REGRESSION AND CAUSAL REGULARIZATION

One of the ultimate aims of science is to establish causal relationships (Pearl, 2009). Inferring causality from observational or heterogeneous data with unspecific interventions is an overly ambitious task and necessarily requires strong untestable assumptions. Regression models, and distributional regression such as GAMLSS, provide a weaker association measure than a causal one between a response variable *Y* and some covariates *X*. However, (distributional) regression models can be regularized toward causality, without claiming to infer causal effects, but leading to a certain kind of invariance, stability, and robustness across experimental settings (Arjovsky et al., 2019; Bühlmann, 2020a, 2020b; Peters et al., 2016; Rothenhäusler et al., 2021). Such additional stability can be very useful for improving generalisability to other settings, and better external validity and statistical replicability of findings.

The idea of causal regularization for enhancing stability and better external validity has been extended to a certain class of distributional regression models (Kook et al., 2022). The file “causal‐regularization‐supplement” in the repository (see end of Section 6), features the application of causal‐regularized distributional regression to the OULAD data set to demonstrate improved worst‐case prediction and better external validity.

## 6 DISCUSSION AND CONCLUSIONS

This article had the goal of overviewing some of the modeling properties allowed by the GAMLSS framework. In order to do so, an open access data set pertinent to LA/EDM was used. The analyses did not intend to make theoretical claims relating to the data set but simply illustrate how GAMLSS could be used for supervised statistical learning of data. It was then argued and showed that GAMLSS are a flexible and interpretable regression‐oriented modeling approach that enables investigating the effect of the covariates on the dependent variable's location, scale, skewness, and kurtosis parameters (more details on GAMLSS in Stasinopoulos et al., 2017; Rigby et al., 2019). The analysis also illustrated that GAMLSS allows building both explanatory and predictive models and producing both types of models is a must in proper statistical learning (Shmueli, 2010; Yarkoni & Westfall, 2017). Likewise, it was shown how distributional regression methods, such as GAMLSS, can be tweaked via causal regularization for inferring causality and thus favoring statistical replicability. The following paragraphs revolve around methodological and statistical issues relating to GAMLSS type analyses and statistical learning in general.

The data were modeled with a GB1 distribution. Although the traditional two‐parameter Beta distribution (BE) did not provide a good fit, it does prevent this distribution to be considered for data modeling. It may well be the case that while the GB1 may not be a good model in a similar data set, the BE could be. A short document found in the repository (see link in the last paragraph) provides mathematical arguments in favor of the Beta distribution. It is also worth mentioning that GAMLSS are not the only way to analyze continuous data. As shown in the Supporting Information, a GAM (with penalisation) analysis is also possible. GAM‐type analysis is contained within the GAMLSS framework and it has been shown to be instrumental in modeling autocorrelations in experimental data (Baayen et al., 2017). Alternatively, robust regression (Ronchetti, 2021; Rousseeuw & Hubert, 2018) and distributional regression methods such as quantile regression (Waldman, 2018) could have been educated choices (see Kneib et al., 2021 for other distributional regression approaches). An interesting related analytical approach is that of scoring rules (Gneiting & Raftery, 2007). Scoring rules enable to evaluate the predictive ability of distributional regression models, but they require the explicit availability of a predictive distribution which is provided by GAMLSS but not by all predictive approaches. Within a multiverse analysis framework (Steegen et al., 2016), performing several valid statistical analyses is indeed encouraged as they enable to determine patterns in data.

Although it was shown herein that the GAMLSS framework can cater for a supervised statistical learning approach to data analysis, it does not prevent mixing GAMLSS with unsupervised learning techniques. For example, classification algorithms such as the “one rule” (a.k.a. 1R, see Holte, 1993, implemented in R via the OneR package) and “Boruta” (Kursa & Rudnicki, 2010, implemented in R via the Boruta package) can be used for variable selection and candidate GAMLSS regression models can be built by combining the best subset of variables. Finally, the resulting models' predictive power could be assessed via cross‐validation (note though that, besides cross‐validation, models should be externally validated). Indeed, there is a recent method called distributional regression forests that blend decision trees (a predictive model popular in ML) and GAMLSS regression (see Schlosser et al., 2019 and the disttree R package). These are approaches worth exploring in silico and through real data sets. Ultimately, the goal is to promote statistical learning and modeling and minimize reliance on hypothesis testing. GAMLSS, and the techniques mentioned above, allow precisely this.

Admittedly, the data set featured in the analyses is not high dimensional (i.e., *p* < *n*); however, GAMLSS can deal with high‐dimensional data (i.e., *p* > *n*) where the estimation of the coefficients via maximum likelihood methods would be intractable. As mentioned above, distributional models can also be fitted using gradient‐based boosting methods (Mayr et al., 2012; Mayr & Hofner, 2018). The boosting estimation approach consists of fitting simple submodels by means of gradient descent. In each iteration only the best fitting independent variable is added to the model. Hence, if a regressor does not improve the model fit, the algorithm retains its partial effect at zero, thus excluding the variable from the model. Thus, the number of fitting iterations becomes the main tuning parameter and it is typically determined using cross‐validation. In short, estimating GAMLSS via gradient‐based boosting carries out data‐driven variable selection, shrinkage of the estimated coefficients, and addresses ill‐posed scenarios such as multicollinearity in the covariates and high dimensionality (*p* > *n*) while retaining interpretability of the estimated partial effects (Hofner et al., 2016). A short tutorial featuring gradient‐based boosting modeling via GAMLSS are available in the Supporting Information.

It is also important to point out that recent developments in GAMLSS methodology allow for the inclusion of unstructured or nontabular data into the distributional model, resulting in “semi‐structured deep distributional regression” (Rügamer et al., 2020). This recent extension of the distributional regression framework combines advancements in ML and statistics that allow statistical modeling of more complex data structures while retaining the interpretability of the fitted model.

Finally, there has been a growing interest in the topic of causality. It was suggested herein that GAMLSS can be modified to exhibit stability and invariance of regression fits; and these are sensible proxies of causality. That is, GAMLSS are a distributional regression framework “geared toward causality” (Bühlmann, 2020a) in that it can be used to examine stabilization of estimated fits across perturbations (in relation to cross‐validation, it is important to note that causal models are not suitable for prediction if there is no distribution shift between training and validation data since including noncausal covariates improves prediction). A recent proposal has demonstrated that combining instrumental variable estimation with GAMLSS is also a fruitful step in this front (Briseño‐Sánchez et al., 2020).

R codes, Supporting Information, and data sets used in the analyses can be found at https://cutt.ly/2WuyxXz.

## AUTHOR CONTRIBUTIONS

**Fernando Marmolejo‐Ramos:** Conceptualization (lead); investigation (lead); methodology (equal); project administration (lead); software (equal); supervision (lead); visualization (equal); writing—original draft (lead); writing—review and editing (lead). **Mauricio Tejo:** Conceptualization (equal); methodology (equal); software (equal). **Marek Brabec:** Conceptualization (equal); methodology (equal); software (equal). **Jakub Kuzilek:** Data curation (lead); formal analysis (equal). **Srecko Joksimovic:** Writing—review and editing (supporting). **Vitomir Kovanovic:** Writing—review and editing (supporting). **Jorge González:** Writing—review and editing (supporting). **Thomas Kneib:** Writing—review and editing (supporting). **Peter Bühlmann:** Methodology (equal); software (lead); writing—original draft (equal); writing—review and editing (equal). **Lucas Kook:** Methodology (equal); software (equal); writing—review and editing (equal). **Guillermo Briseño‐Sánchez:** Methodology (equal); software (equal); writing—review and editing (supporting). **Raydonal Ospina:** Conceptualization (equal); data curation (equal); formal analysis (lead); methodology (lead); software (equal); writing—original draft (equal); writing—review and editing (equal).

## CONFLICT OF INTEREST

The authors have declared no conflicts of interest for this article.

## RELATED WIREs ARTICLE

Educational data mining and learning analytics: An updated survey

## Data Availability

we provide a link to an OA repository where data and R codes are available

## ENDNOTES

^1^
In this article, LA and EDM are referred to indistinctly.

^2^
The term “effect” is commonplace in the regression literature (i.e., main effects, interaction effects, fixed effects, and random effects; see chapter 3 in James et al., 2017; Sheskin, 2011) and it stands for relationships between the independent variables and the dependent variable. However, the term “effect” can be interpreted as “cause” only under certain study designs (e.g., chapter 18 in Gelman et al., 2020) or when instrumental variable techniques are used (see section 2.3 in Bell et al., 2019).

^3^
For many practical situations and computational implementations, four parameters are required to determine the distribution *f*(*y*_*i*_|(*θ*_*i*1_, *θ*_*i*2_, …, *θ*_*ip*_)). The *R* implementation denotes these parameters as *μ*_*i*_ = *θ*_*i*1_, *σ*_*i*_ = *θ*_*i*2_, *ν*_*i*_ = *θ*_*i*3_, *τ*_*i*_ = *θ*_*i*4_, for *i* = 1, …, *n*. The first parameter is location (usually the mean or median), the second is scale (usually the standard deviation or precision), and the others are shape parameters (e.g., skewness and kurtosis). For computational implementations of GAMLSS it is desirable that the probability density of *y* and its first derivatives with respect to each of the parameters must be computable. Also, when the covariates are stochastic (useful in ML for investigating causal relation between variables) the density *f*(*y*_*i*_| ***θ*** ^*i*^) is taken to be conditional on their values.

^4^
A simple and informative metric that combines location and scale is the coefficient of variation (see Arachchige et al., 2022; Ospina & Marmolejo‐Ramos, 2019). This measure of relative dispersion is given, in its classic form, by the ratio of the standard deviation to the mean (i.e., σμ). This metric is rather underused and just recently has been revived in the fields of data mining and machine learning (Bindu et al. (2020)).

^5^
Although statistical analyses and hypotheses tests are traditionally performed on the data's location parameter (e.g., mean or median differences between treatment groups), it is less common to perform tests and analyses on scale parameters (e.g., differences between groups' variances) and even more uncommon on shape parameters (i.e., skewness and kurtosis. In some fields, though, these parameters are investigated; see Ben‐David et al., 2015). One reason for this situation is that the interpretation of the shape parameters is challenging due to lack of agreement on what they represent (e.g., kurtosis, traditionally understood as data's “peakedness,” has been defined as an index of data's propensity to outliers; see Westfall, 2014). Another reason is that for quality estimation of more delicate features (like higher order moments, various shape parameters) vast amounts of data are needed and that are traditionally not available. Nowadays, big data are increasingly common, allowing for analyses that go far beyond traditional viewpoints and the OULAD data set is just one example

^6^
The degrees of freedom is a useful concept for describing model complexity and it is asymptotically equal to the trace of the usual “hat” matrix plus the number of parameters in the error covariance matrix of the model (Hastie & Tibshirani, 1990).

^7^
The probability density function of the GB1 is $f\left(y|\mu ,\sigma \nu ,\tau \right)=\frac{{\tau \nu }^{\beta }y^{\tau \alpha -1}{\left(1-y^{\tau }\right)}^{\beta -1}}{B\left(\alpha ,\beta \right){\left[\nu +\left(1-\nu \right)y^{\tau }\right]}^{\alpha +\beta }}$, where 0 < *y* < 1, *α* = *μ*(1 − *σ* ^2^)/*σ* ^2^ and *β* = (1 − *μ*)(1 − *σ* ^2^)/*σ* ^2^, and *α* > 0, *β* > 0. The location is *μ* = *α*/(*α* + *β*) and the scale is *σ* = (*α* + *β* + 1)^−1/2^.

^8^
By default, gamlss() chooses the category that comes first alphabetically or numerically (alphanumerically) as the reference category. In this case, Female is the reference of the variable gender and A Level or Equivalent is the baseline of the variable highest education. By using the *R* function relevel() it is possible to change the reference category for each factor variable.

^9^
Note that these are all mean‐based (point‐prediction performance) measures commonly used in ML work. As the predictive power of GAMLSS is better assessed via out‐of‐sample log‐likelihood, it is thus not surprising that GAMLSS did not outperform the other methods under these three metrics.
